# Modulation of Measles Virus N_TAIL_ Interactions through Fuzziness and Sequence Features of Disordered Binding Sites

**DOI:** 10.3390/biom9010008

**Published:** 2018-12-27

**Authors:** Christophe Bignon, Francesca Troilo, Stefano Gianni, Sonia Longhi

**Affiliations:** 1 CNRS and Aix-Marseille Univ Laboratoire Architecture et Fonction des Macromolecules Biologiques (AFMB), UMR 7257 Marseille, France; francesca.troilo@uniroma1.it; 2 Istituto Pasteur—Fondazione Cenci Bolognetti, Dipartimento di Scienze Biochimiche ‘A. Rossi Fanelli’ and Istituto di Biologia e Patologia Molecolari del Consiglio Nazionale delle Ricerche, Sapienza Università di Roma, 00185 Rome, Italy; stefano.gianni@uniroma1.it

**Keywords:** IDP, fuzzy interactions, protein complementation assays, split-GFP reassembly, kinetics

## Abstract

In this paper we review our recent findings on the different interaction mechanisms of the C-terminal domain of the nucleoprotein (N) of measles virus (MeV) N_TAIL_, a model viral intrinsically disordered protein (IDP), with two of its known binding partners, i.e., the C-terminal X domain of the phosphoprotein of MeV XD (a globular viral protein) and the heat-shock protein 70 hsp70 (a globular cellular protein). The N_TAIL_ binds both XD and hsp70 via a molecular recognition element (MoRE) that is flanked by two fuzzy regions. The long (85 residues) N-terminal fuzzy region is a natural dampener of the interaction with both XD and hsp70. In the case of binding to XD, the N-terminal fuzzy appendage of N_TAIL_ reduces the rate of α-helical folding of the MoRE. The dampening effect of the fuzzy appendage on XD and hsp70 binding depends on the length and fuzziness of the N-terminal region. Despite this similarity, N_TAIL_ binding to XD and hsp70 appears to rely on completely different requirements. Almost any mutation within the MoRE decreases XD binding, whereas many of them increase the binding to hsp70. In addition, XD binding is very sensitive to the α-helical state of the MoRE, whereas hsp70 is not. Thus, contrary to hsp70, XD binding appears to be strictly dependent on the wild-type primary and secondary structure of the MoRE.

## 1. Structural Properties and Molecular Partnership of N_TAIL_

**Figure 1:**
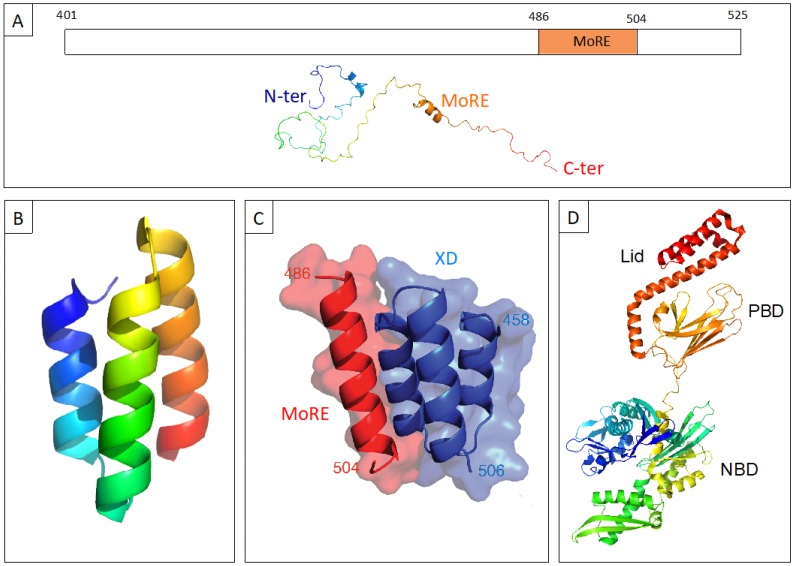
(**A**) Schematic representation of the C-terminal domain of the nucleoprotein (N) of measles virus (MeV) N_TAIL_ (upper panel) and cartoon representation of an N_TAIL_ conformer generated using Flexible-Mecano [49]. (**B**) Ribbon representation of the crystal structure of the C-terminal X domain of the phosphoprotein of MeV XD (PDB code 1OKS). (**C**) The structure of the chimeric construct made of MeV XD (blue) and of the molecular recognition element MoRE of N_TAIL_ (red) (PDB code 1T6O). (**D**) Cartoon representation of the crystal structure of hsp70 based on PDB codes 1HJO and 4JNF. The relative orientation of the two hsp70 domains (i.e., amino acids 3 to 382 and amino acids 389 to 610) is based on the structure of a form encompassing residues 1 to 554 (PDB code 1YUW). The three constituent domains of hsp70, i.e., nucleotide binding domain (NBD, aa 1 to 384), peptide binding domain (PBD, aa 384 to 543) and “lid” (aa 543 to 641) (see [18] and references therein cited) are highlighted.

The nucleoprotein (N) of measles virus (MeV) consists in a large structured moiety (N_CORE_, aa 1 to 400) and in a C-terminal domain (N_TAIL_, aa 401 to 525 of N) that is intrinsically disordered [1] (Figure 1A). The N_TAIL_ protrudes from the globular core of N and is exposed at the surface of the viral nucleocapsid [2,3,4,5,6]. The latter is made of a regular array of N monomers wrapping the RNA genome into a helicoidal arrangement. The exposure of N_TAIL_ at the surface of the nucleocapsid allows recruitment of the phosphoprotein (P) via interaction with the C-terminal X domain (XD) of the latter [7,8,9,10]. The phosphoprotein (P) is required for both transcription and replication, as it tethers the viral Large protein (L), which possesses all the enzymatic activities required for RNA synthesis, onto the nucleocapsid template (for a review see [11]).

Structural disorder is known to be a determinant of protein interactivity: the enhanced plasticity of intrinsically disordered proteins (IDPs) and regions (IDRs) allows for the enlargement of their molecular partnership [12,13,14]. In line with this, MeV N_TAIL_ binds to numerous partners. Beyond the X domain of the P protein, N_TAIL_ also interacts with the viral matrix protein [15]. In addition, it also interacts with host proteins, such as the major inducible heat shock protein 70 (hsp70) [16,17,18], a nuclear export protein [19], the interferon regulatory factor 3 [20,21], a cell receptor involved in MeV-induced immunosuppression [22,23], peroxiredoxin 1 [24], and proteins of the cell cytoskeleton [25,26].

The N_TAIL_ and XD proteins interact with each other forming a 1:1 stoichiometric complex with an equilibrium dissociation constant (K_D_) in the μM range [27,28]. The crystal structure of MeV XD has revealed that this domain consists of a bundle of three antiparallel α-helices [9,10,29] (Figure 1B). In solution however, two distinct structural forms differing in their degree of compactness coexist [30,31].

The structural arrangement of XD in a triple α-helical bundle, as well as the disordered nature of N_TAIL_ [32], are also conserved in the related Nipah and Hendra viruses, whose N_TAIL_-XD complexes are similar to that of MeV [27,33]. Binding to XD triggers α-helical folding of a short N_TAIL_ region (Box2, aa 486 to 504 of MeV N, and Box3, aa 473 to 493 of *Henipavirus* N), referred to as a Molecular Recognition Element or MoRE [7,9,10,27] (Figure 1A). The MoREs are short, transiently populated secondary structures within IDRs that are often structurally biased towards their bound state [34]. The crystal structure of a MeV chimeric construct in which XD is covalently attached to the MoRE of N_TAIL_ (aa 486 to 504) was solved at 1.8 Å [10]. The structure consists of a pseudo-four helix complex in which the MoRE of N_TAIL_ adopts a parallel orientation with respect to XD and is embedded in a large hydrophobic cleft delimited by XD helices α2 and α3 [10] (Figure 1C).

The MoRE is partly preconfigured as an α-helix in the absence of XD in both MeV and henipaviruses [5,29,33,35,36,37,38]. This partial pre-configuration facilitates the folding-upon-binding process by rendering the structural transition to the (partially) folded conformation energetically less demanding [34]. In spite of this pre-configuration, N_TAIL_ was shown to fold according to a folding-after-binding mechanism [28,33,39,40].

Mutational studies coupled to Φ-value analysis led to a detailed structural description of the folding and binding events occurring in the recognition between MeV N_TAIL_ and XD [41]. Analysis of the impact of single-amino acid substitutions in N_TAIL_ on the reaction mechanism allowed the identification of key residues involved in the initial recognition between N_TAIL_ and XD, and enabled unraveling of the general features of the folding pathway of N_TAIL_. In addition, analysis of the changes in stability of all the variants revealed that a few substitutions favor the folding step, which highlighted the inherent poor folding efficiency of N_TAIL_, a property that we proposed that could arise from the weakly funneled nature of the energy landscape of IDPs in their unbound state that might dictate a considerable structural heterogeneity (or structural frustration) of the bound state [41].

In both MeV and henipaviruses, following binding to XD, most of N_TAIL_ remains disordered and does not establish stable contacts with XD [8,27,29,33,35,36,37,38,42,43,44]. These N_TAIL_-XD complexes are therefore illustrative examples of fuzziness [45]. Fuzziness may confer various functional advantages, such as the ability to interact with alternative partners and/or to establish simultaneous interactions with different partners. Fuzziness also provides a way to reduce the entropic penalty that accompanies the disorder-to-order transition, thereby leading to enhanced affinity. Tuning fuzziness therefore constitutes an additional manner by which IDPs can modulate the interaction strength with their partners. Furthermore, disordered appendages can harbor regulatory post-translational modification sites, can serve for partner fishing via non-specific, transient contacts, and can accommodate binding sites for additional partners [46,47,48].

In line with these abilities, the C-terminal fuzzy region of MeV N_TAIL_ encompassing residues 517 to 525 was shown to serve as a low-affinity binding site for hsp70 [17,18], a large cellular protein with a markedly different structural organization (Figure 1D) with respect to XD. The heat shock protein 70 (hsp 70) was shown to stimulate both viral transcription and replication, with this ability relying on interaction with N_TAIL_ [16,17,50,51,52,53,54,55]. Binding experiments showed that the major hsp70-binding site is however located within Box2 [56]. Since hsp70 was found to competitively inhibit the binding of XD to N_TAIL_ [17], it has been proposed that hsp70 could enhance viral transcription and replication by destabilizing the P–N_TAIL_ interaction, thereby promoting successive cycles of binding and release that are essential for the polymerase to progress along the nucleocapsid template [8,17]. The hsp70-dependent reduction of the stability of P–N_TAIL_ complexes would thus rely on competition between hsp70 and XD for binding to the α-MoRE of N_TAIL_, with recruitment of hsp70 being ensured by both Box2 and Box3 [17]. Although the hsp70-binding site(s) within N_TAIL_ have been mapped, no structural information on the complex is available.

In the following sections we summarize available data pertaining to the impact of the long, N-terminal fuzzy appendage of N_TAIL_ on binding to both XD and hsp70. We also summarize the available molecular information on the sequence and secondary structure requirements for N_TAIL_-XD and N_TAIL_-hsp70 binding. Altogether, these studies contribute to enlarge our knowledge of the molecular determinants underlying the ability of hsp70 to interact with N_TAIL_ and, more generally, add “another brick to the wall” towards the ambitious goal of building up a comprehensive understanding of the mechanisms by which IDPs recognize their partners.

## 2. The N-Terminal Fuzzy Region of N_TAIL_ down Regulates the Binding of the MoRE to Both XD and Hsp70

**Figure 2:**
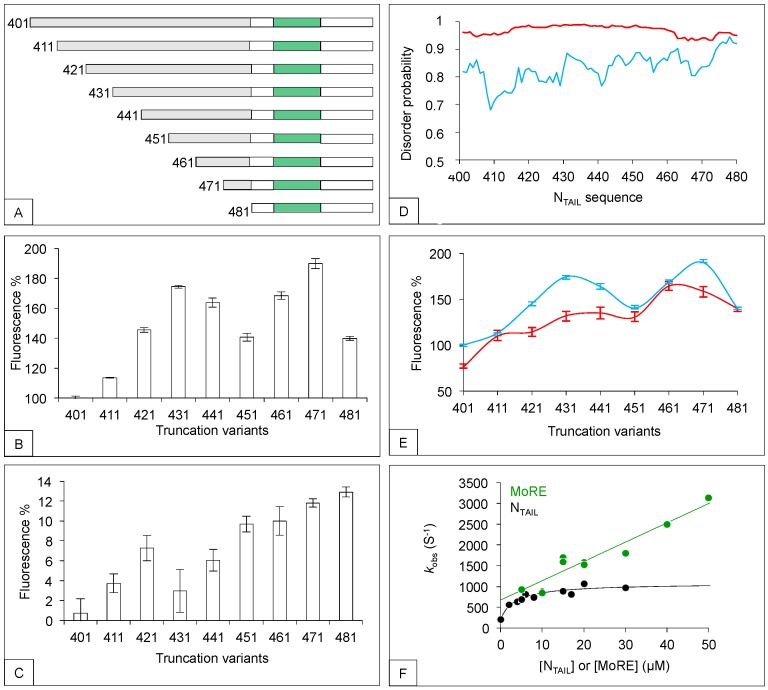
Effect of the N-terminal fuzzy region of N_TAIL_ on XD and hsp70 binding. (**A**) N_TAIL_ deletion variants were generated as described [60]. The N-terminal residue is indicated. The N-terminal fuzzy region subjected to truncation is shown in grey and the MoRE is shown in green. (**B**,**C**) Split- green fluorescent protein (GFP) complementation assay using XD (**B**) and hsp70 (**C**). Shown are the mean values and standard deviation (SD) of an experiment performed in triplicate. Results are expressed as percentage with 100% being the fluorescence value provided by full-length N_TAIL_ (401). For a detailed description of the procedure see Supplementary Information. (**D**) IUPred [64] disorder prediction of wtN_TAIL_ (blue) and artN_TAIL_ (red) from residue 401 to residue 480. (**E**) Fluorescence values obtained by split-GFP complementation assays using wild type (wt) (blue line) and art (red line) N_TAIL_ truncation variants and XD. Shown are the mean values and SD of an experiment performed in triplicate. Results are expressed as percentage with 100% being the fluorescence value provided by full-length wtN_TAIL_ (401). (**F**) Binding kinetics of XD (at a constant concentration of 2 μM) with excess concentrations of either wtNTAIL (black circles) or a peptide mimicking the MoRE (green circles) in 10 mM sodium phosphate buffer and 150 mM NaCl at pH 7.0. Under all conditions, there was an at least fivefold difference in concentration between the two proteins to ensure pseudo-first order conditions. Experiments were carried out using a PTJ-64 capacitor-discharge T-jump apparatus (Hi-Tech, Salisbury, UK). The temperature was rapidly changed with a jump size of 9 °C, from 11 °C to 20 °C. Data were taken from [60].

As recalled in the introduction, the MoRE of N_TAIL_ (aa 486 to 504) is responsible for XD binding and is preceded by a long, N-terminal fuzzy region (aa 401 to 488). We have investigated the role of this region by shortening it by ten residue intervals from aa 401 to aa 481 (Figure 2A), and then assessing the binding ability of each truncation variant using a split-green fluorescent protein (GFP) complementation assay [57,58]. In this assay, two proteins (X and Y) known to interact with each other are respectively fused to the C-terminal end of the first seven N-terminal moiety of GFP (NGFP) and the N-terminal end of the last four β-strands C-terminal moiety of GFP (CGFP) of GFP. Separately, NGFP-X and Y-CGFP are unable to fluoresce. However, when NGFP-X and Y-CGFP are co-expressed in *E. coli*, X and Y bind to each other within the cell, allowing NGFP and CGFP to reconstitute the full-length fluorescent GFP. Since the fluorescence is proportional to the affinity between X and Y [59,60], the interaction between different combinations of NGFP-X and Y-CGFP can be compared by simply measuring the fluorescence of the bacteria co-expressing NGFP-X and Y-CGFP. In our case, X was N_TAIL_ or its truncation variants and Y was either XD or hsp70.

Results show a non-monotonic fluorescence increase with the truncation, with both XD (Figure 2B) and hsp70 (Figure 2C). In agreement with the known higher affinity of N_TAIL_ for XD (3 μM) [28] compared to that for hsp70 (70 μM) [18], the overall fluorescence was found to be higher for XD than for hsp70 (see the different Y-axis scales between Figure 2B,C). Thus, the fuzzy N-terminal region of N_TAIL_ negatively regulates the binding of N_TAIL_ to two partners that differ in both size and affinity. We have obtained similar results when N_TAIL_ and XD from NiV and HeV were used [61] or when another protein complementation assay based on split-luciferase [62] was used. Thus, the negative effect of the fuzzy N-terminal region of N_TAIL_ on XD binding is shared by at least three paramyxoviruses and is maintained irrespective of whether the assay generates reversible (luciferase) or irreversible (GFP) complexes [61].

We sought possible reasons for this negative effect. The importance of the primary structure of N_TAIL_ N-terminal region was first assessed. Since this region remains disordered after binding, a possible reason for its observed negative effect on binding could be its mere fuzziness. If this were the case, then swapping the wild-type sequence with another unrelated sequence would be expected to elicit similar effects provided that it is similarly disordered. To test this hypothesis, we replaced the wild-type N-terminal fuzzy region of N_TAIL_ (aa 401 to 480) with another non-natural sequence. Compared to its wild-type counterpart, this artificial sequence (i) has the same number of residues, (ii) is predicted to be slightly more disordered (Figure 2D), (iii) shares only 6% identity. This artificial sequence was fused to the remaining part (aa 481 to 525) of wild-type N_TAIL_ to reconstitute an artificial full-length N_TAIL_ (aa 401 to 525) (artN_TAIL_). We then generated the same series of truncation variants as those previously generated from the wild-type sequence (wtN_TAIL_) (Figure 2A) and compared their effect on the binding to XD. As shown in Figure 2E, wtN_TAIL_ and artN_TAIL_ truncation variants yielded similar binding patterns, with the binding strength increasing non-monotonically with the truncation. However, results were not identical. Compared to wtN_TAIL_, the profile obtained with artN_TAIL_ was more linear, and each artN_TAIL_ variant displayed a slightly lower interaction strength towards XD than its wild-type counterpart, a property that could be related to the higher disorder probability of full-length artN_TAIL_ (Figure 2D). Thus, the negative effect of N_TAIL_ N-terminal fuzzy region (aa 401 to 485) on XD binding was not due to its specific sequence but to a combination of length and fuzziness. The sequence-independent nature of the effect exerted by the disordered appendage is not unique to N_TAIL_, having also been observed in the case of human UDP-α-D-glucose-6-dehydrogenase. This enzyme possesses a C-terminal disordered region that entropically rectifies the dynamics and structure of the enzyme to favor binding of an allosteric inhibitor, with this effect being independent from both primary structure and chemical composition [63].

We tried to perform the same experiments using hsp70, but got results suffering from low reproducibility for unknown reasons (not illustrated). We further investigated the molecular mechanisms by which the fuzzy appendage of MeV N_TAIL_ influences the interaction with XD by analyzing binding kinetics (Figure 2F). In the case of full-length N_TAIL_ (aa 401–525), a hyperbolic dependence of *k_obs_* (the macroscopic observed rate constant) on ligand concentration was observed, which accounts for the folding of N_TAIL_ becoming rate-limiting at high reactant concentrations [28]. Conversely, when a MoRE-mimicking peptide (aa 485 to 506) was used, linear kinetics was observed. Kinetic experiments could not be performed using hsp70 because of the low affinity of the interaction, and due to the presence of numerous tryptophan residues that could jeopardize the analysis. In conclusion, the N_TAIL_ N-terminal region could dampen the N_TAIL_/XD interaction, at least in part, by lowering the rate of folding of the MoRE, although the subtle mechanisms underlying this ability remain elusive and await future studies to be unraveled.

## 3. The Bindings of XD and Hsp70 to N_TAIL_ MoRE Rely on Different Primary and Secondary Structure Requirements

We have seen that MeV N_TAIL_ N-terminal region (401 to 485) has comparable negative effects on the binding of two different N_TAIL_ partners: XD, a small viral protein [9] with a relatively high affinity (3 μM) [28] and hsp70, a large cellular protein with a lower affinity (70 μM) for N_TAIL_ [18]. Although the MoRE has been shown to be the major hsp70-binding site [17,18], the structure of N_TAIL_-hsp70 complex has not been solved yet contrary to the N_TAIL_-XD complex [10]. As a consequence, we do not know whether the MoRE folds into an α-helix upon binding to hsp70 as it does upon XD binding and whether the interaction relies on the same MoRE residues. The relevance of investigating the molecular mechanisms governing the N_TAIL_/hsp70 interaction lies in its well-documented impact on viral transcription and replication [16,17] and on the innate immune response [65].

### 3.1. Sequence Requirements of N_TAIL_ Molecular Recognition Element for XD and Hsp70 Binding

**Figure 3:**
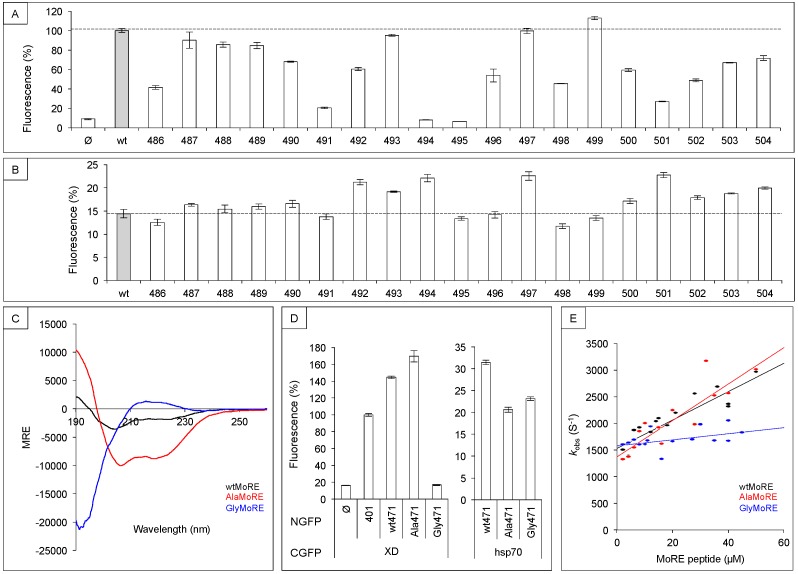
Effect of primary and secondary structures of the MoRE on XD and hsp70 binding. (**A**,**B**) Alanine scanning mutagenesis of N_TAIL_ MoRE. MoRE residues (aa 486 to 504) of MeV N_TAIL_ truncation variant 471 were individually mutated into an alanine (or a glycine when the wild-type residue was an alanine). The binding ability of each single N_TAIL_ variant was then compared to that of wild-type N_TAIL_ by split-GFP complementation assay using either XD (**A**) or hsp70 (**B**). Ø, negative control (fluorescence background obtained using an empty vector encoding NGFP alone); wt, positive control (i.e., wild-type truncation variant 471). Results are expressed as percentage with 100% being the fluorescence value provided by wt truncation variant 471. The horizontal dotted line indicates the binding of the positive control. (**C**) Far-UV circular dichroism spectra of wtMoRE, AlaMoRE, and GlyMoRE peptides. (**D**) Fluorescence values obtained by split-GFP complementation assays using N_TAIL_ MoRE variants with different α-helicities. See A for details; 401, full-length wtN_TAIL_; wt471, 471 truncated variant with a wtMoRE; Ala471, 471 truncated variant with AlaMoRE; Gly471, 471 truncated variant with GlyMoRE. (**E**) Binding kinetics of MoRE peptides to XD. Data shown in panels A, B, and D are the mean values and SD of an experiment performed in triplicate. Data were taken from [66].

To gain insights into this biologically relevant question, we first alanine scanned the MoRE, and assessed the effect of these substitutions by monitoring the binding of each individual variant to XD and hsp70 using the split-GFP complementation assay [64]. We used N_TAIL_ truncation variant 471 (aa 471 to 525) as backbone to derive single-site variants because it binds XD better than full-length N_TAIL_ (Figure 2B) [61], and therefore provides higher fluorescence signals in split-GFP complementation assay that are more appropriate than weak signals to study subtle modulation effects. In the case of XD binding (Figure 3A), most alanine variants exhibited a decreased binding compared to that of the wild-type sequence and, in a few cases (residues Ser491, Ala494, Leu495, Met501), the single alanine (or glycine) substitution essentially abrogates binding [66]. These latter residues can therefore be defined as critical for XD binding, a conclusion in agreement with the 3D structure of the MeV MoRE-XD complex in which all these residues point toward XD and not to the solvent [10]. Very different results were obtained with hsp70 (Figure 3B) [64]. First, several variants exhibited an increased binding compared to the wild-type sequence. Secondly, no single residue proved to be mandatory for binding to hsp70. Thus, although N_TAIL_ binding to both XD and hsp70 was down-regulated by the N_TAIL_ N-terminal fuzzy region (Figure 2), these two proteins bind the MoRE using different residues thereof, and hence through different mechanisms.

**Figure 4:**
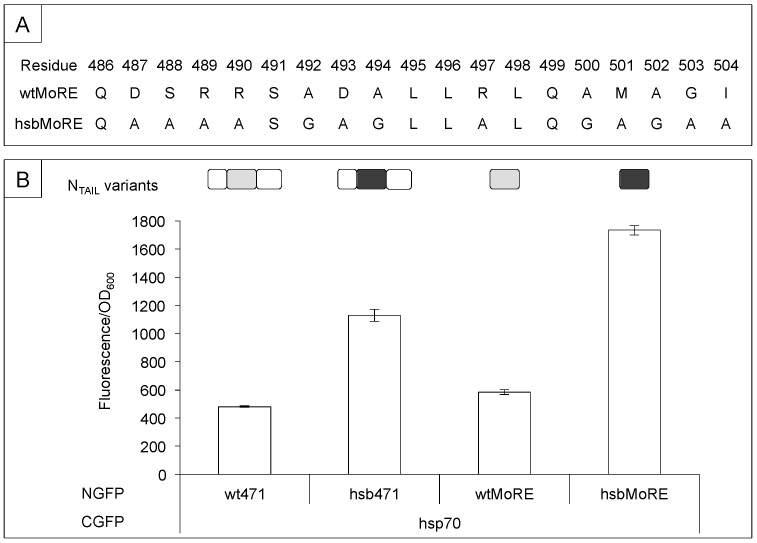
Binding abilities of hsb towards hsp70. (**A**) Amino acid sequence of wt and hsbMore. In the latter, all the residues individually shown to lead to increased N_TAIL_-hsp70 binding strength by the alanine scanning mutagenesis were collectively replaced with alanine, or with glycine when the wild-type residue was alanine. (**B**) Binding abilities of N_TAIL_ variants as obtained by split-GFP complementation assays. *Y*-axis: fluorescence values of each culture divided by the optical density at 600 nm. *X*-axis: N_TAIL_ variants-hsp70 pairs. Shown are the mean values and SD of an experiment performed in triplicate. The scheme of the N_TAIL_ constructs is shown above the graph, with wt and hsb MoREs being represented in grey and black, respectively, and fuzzy regions in white. Orientation is from left (N-terminal end) to right (C-terminal end). Data were taken from [66].

Based on the results provided by the alanine-scanning mutagenesis, we conceived an hsp70 “super binder” (hsb) that was obtained by collectively introducing all the substitutions that individually increased the binding to hsp70 (see Figure 4A) in the context of truncated variant 471 (hsb471). This rationally designed variant displayed a much higher binding strength (2.35 times) towards full-length hsp70 than wt471 in a split-GFP complementation assay (compare wt471 to hsb471 and wtMoRE to hsbMoRE in Figure 4B). Because of the dampening effect of the N-terminal fuzzy appendage (Figure 2C), this enhancement in affinity was even more pronounced when hsbMoRE was used alone rather than in the context of truncated variant 471 (compare hsb471 and hsbMoRE bindings in Figure 4B).

The three-fold increase in binding strength towards hsp70 upon replacement of as many as 13 residues out of 19 (i.e., almost 70%) of the sequence of the wtMoRE with alanine or glycine (Figure 4A) is puzzling. How can N_TAIL_ binding to hsp70 be specific of the MoRE while being relatively independent of the sequence of the latter? Conceivably, hsp70 may recognize not a precise amino acid sequence or motif, but rather a set of few residues with specific chemical features and no strict positional conservation. While hydrophobicity on its own cannot explain the increased binding strength of hsbMoRE [64], the enrichment in Ala, Gly, and Leu residues (in this order) and the depletion in Asp residues of hsbMoRE (Figure 4A) might provide a rational explanation: indeed, previous studies identified these features as favoring binding of peptides to hsp33, a redox-regulated chaperone [67].

### 3.2. Secondary Structure Requirements of N_TAIL_ Molecular Recognition Element for XD and Hsp70 Binding

Single residue substitutions of the alanine scanning aimed at providing information on the sequence requirement of XD and hsp70 binding but not at changing the secondary structure of the MoRE. The latter is known to fold into an α-helix upon XD binding. However, nothing is known about the conformation it takes upon binding to hsp70. To address this question, we constructed two MoRE variants with opposite folding properties [66]. Both MoRE variants were generated using truncation variant 471 as backbone for the reason given above. In the first one (Ala471), all residues the alanine scanning identified as non-critical for XD binding were replaced with alanine. In the second one (Gly471), those residues were replaced with glycine. Since alanine promotes α-helix formation whereas glycine has the opposite effect [68], Ala471 and Gly471 were expected to be more and less α-helical than wtMoRE, respectively. This assumption, strengthened by disorder prediction and modeling [66], was experimentally confirmed by circular dichroism (CD) analysis of MoRE peptides (Figure 3C).

The wt471, Ala471, and Gly471 variants were then tested for their ability to bind XD or hsp70 by split-GFP complementation assay. Results (Figure 3D, left panel), indicated that increasing the α-helicity (Ala471) slightly increased binding to XD compared to wt471, whereas the lack of α-helicity (Gly471) resulted in a complete loss of binding in spite of the presence of the residues revealed to be critical for XD binding by alanine scanning [64]. Conversely, Ala471 and Gly471 behaved similarly when assessed for their binding to hsp70: they both exhibited a moderately decreased binding compared to wt471 (Figure 3D, right panel) [66]. The lower XD binding ability of Gly471 compared to that of wt471 and Ala471 was also confirmed by kinetics experiments (Figure 3E). While AlaMoRE and wtMoRE behaved similarly, there was a detectable destabilization of the complex in the case of GlyMoRE as judged from the lower slope of its binding kinetics [64]. These results definitely indicate that XD and hsp70 did not rely on the same structural requirements to bind to the MoRE of N_TAIL_. More specifically, increasing the α-helicity of the MoRE increased XD binding but decreased hsp70 binding suggesting that the latter did not trigger α-helical folding of the MoRE, a conclusion strengthened by the ability of hsp70 to bind a MoRE that is unable to fold into an α-helix.

In conclusion, in addition to using a different set of N_TAIL_ residues, XD and hsp70 did not induce the same folding within the MoRE, therefore indicating that they likely interacted with N_TAIL_ through completely different mechanisms.

## 4. Conclusions

Deletion studies have shown that the long, N-terminal fuzzy region of N_TAIL_ inhibits the interaction with XD and hsp70. This raises the question of what could be the possible functional role of this auto-inhibition. According to the so-called cartwheeling mechanism, the N_TAIL_-XD interaction needs to be dynamically made and broken to ensure progression of the polymerase complex onto the nucleocapsid to allow transcription and replication [69]. A too strong interaction between N_TAIL_ and XD is therefore predicted to hinder the polymerase processivity. The discovery that the fuzzy appendage acts as a natural dampener of the interaction provides a conceptual framework to understand why the MoRE is preceded by such a long arm. It is tempting to speculate that in the course of evolution, the length of this region has been under selective pressure so as to ensure an optimal affinity towards XD. This speculation is in agreement with recent studies by the group of Plemper that showed that a mutated measles virus in which the region preceding the MoRE has been shortened suffers from an imbalance between transcription and replication [70].

Alanine-scanning mutagenesis of the MoRE unveiled that XD is very sensitive to substitutions, in line with experimental evidence showing that the MoRE of N_TAIL_ is poorly evolvable in terms of XD binding [58]. This implies that the sequence of the MoRE has been shaped during evolution to achieve maximal binding to XD, a finding in striking contrast with the postulated positive selection of a long fuzzy appendage dampening the interaction. Although apparently contradictory, these effects of natural selection have in fact resulted in a finely tuned system in which the strongest possible MoRE-XD interaction is “entropically rectified” [63] by the N-terminal fuzzy region of N_TAIL_ to achieve a precise N_TAIL_-XD interaction strength. The latter is in fact required to ensure dynamic anchoring of the L–P polymerase complex [71] and efficient transcription re-initiation at each intergenic junction of the MeV genome [72].

By contrast, hsp70 is much more tolerant to substitutions within the MoRE, and the MoRE-hsp70 interaction appears to be highly evolvable. The high evolvability of the N_TAIL_-hsp70 interaction might arise from the fact that the two binding partners have not been subjected to an as tight co-evolution as that of the N_TAIL_-XD pair due to the multiple functional roles that hsp70 plays in the cell and that are not exclusively related to MeV infection. In addition, a high affinity between N_TAIL_ and hsp70 might not be required for the interaction to take place and elicit the known effects on viral transcription and replication [17,73] and on the innate immune response [65] given the very high intracellular concentrations of both hsp70 and N in MeV infected cells [53]. A high affinity could even be deleterious for the viral replication since hsp70 could then fully out compete XD for N_TAIL_ binding [17].

The discovery that the N_TAIL_-hsp70 interaction does not rely on the same residues mediating the N_TAIL_-XD interaction, and does not imply α-helical folding emphasizes the plasticity and polymorphism of this IDP. The structure adopted in the bound form seems therefore to be “sculpted” by the partner, thereby providing an additional example of “templated folding” [41]. This high extent of malleability with respect to the partner challenges the role of preconfiguration of MoREs in the recognition process. N_TAIL_ seems indeed to be relatively insensitive to the structure of its pre-recognition motif, being able to adopt a non α-helical conformation upon binding to hsp70 in spite of the partial α-helical preconfiguration of its MoRE.

Finally, and from a more applied perspective, the much higher affinity of hsb compared to wt MoRE towards hsp70 holds promise for future potential therapeutic applications. Since the N_TAIL_-hsp70 interaction stimulates viral transcription and replication [16,17], and since hsbMoRE binds hsp70 three times better than wtMoRE, over-expressing hsbMoRE in MeV-infected cells might expectedly inhibit MeV replication (provided that hsbMoRE is non-toxic for eukaryotic cells). Incidentally, hsbMoRE could also be used as an anti-cancer drug, based on previous studies that have described the anti-viral [74] and anti-cancer [75,76] effect of 2-phenylethynesulfonamide, a specific hsp70 inhibitor. Experiments are ongoing in our lab to assess the therapeutic potential of hsbMoRE.

## Supplementary Materials

The following are available online at http://www.mdpi.com/2218-273X/9/1/8/s1.
