# Supplementary material for: Modulation of Measles Virus NTAIL Interactions through Fuzziness and Sequence Features of Disordered Binding Sites
Source: Biomolecules. 2018 Dec 27;9(1):8. doi: 10.3390/biom9010008 (PMC6359293; doi:10.3390/biom9010008)
Supplement: Supplementary file 1 [file biomolecules-09-00008-s001.pdf]

Supplementary Information

Modulation of measles virus NTAIL interactions through fuzziness and sequence features of disordered binding sites

Christophe Bignon [1,*], Francesca Troilo [1,2], Stefano Gianni [2] and Sonia Longhi [1,*]

[1] CNRS and Aix-Marseille Univ, Laboratoire Architecture et Fonction des Macromolecules Biologiques (AFMB), UMR 7257, Marseille, France

[2] Istituto Pasteur – Fondazione Cenci Bolognetti, Dipartimento di Scienze Biochimiche 'A. Rossi Fanelli' and Istituto di Biologia e Patologia Molecolari del Consiglio Nazionale delle Ricerche, Sapienza Università di Roma, Rome, Italy

[*] To whom correspondence should be addressed.

Materials and Methods

Split-GFP complementation assay

E. coli T7-pRos (*i.e.,* T7 cells (New England Biolabs) bearing the pLysS plasmid from Rosetta(DE3) pLysS cells (Novagen)) were co-transformed with plasmid pNGG bearing the coding sequence of either full-length NTAIL (401) or its truncation variants (411 to 481), and plasmid pMRBAD-link-CGFP bearing the coding sequence of either XD or hsp70, and then plated on ampicillin- and kanamycin-containing agar plates. The next day, several colonies were scraped off the plate and used to seed 4 ml of Luria Bertani medium containing ampicillin (100 µg/ml), kanamycin (50 µg/ml), and chloramphenicol (34 µg/ml) in 24-wells deep-wells. Chloramphenicol was used to maintain the pRos plasmid. After one night at 37°C under 200 rpm shaking, pre-cultures were used to seed 3 wells of 24-wells deep-wells with 300 µl of each pre-culture per well and each well containing 4 ml of terrific broth with the same three antibiotics. Cells were grown for an additional 2.5 hours at 37°C. IPTG and arabinose were then added to each culture well at the respective final concentrations of 0.5 mM and 2%, and the deep-wells were incubated over night at 17°C under shaking. The next day, the deep-wells were spun for 5′ at 4000 g in a swinging rotor and the supernatant was discarded. Cell pellets were resuspended in 1 ml of PBS and 10 µl of each cell suspension were diluted in 100 µl PBS in each well of a 96-wells clear bottom black microplate. The optical density at 600 nm (i.e., the number of cells) (OD_{600}) and GFP fluorescence (Fluo) of each well were measured using a TECAN GENios Plus spectrofluorimeter. The fluorescence value of each well was divided by the OD_{600} of the same well (Fluo/OD_{600} ratio), and the mean value of each triplicate ratio and standard deviation were calculated. Results were expressed either as Fluo/OD_{600} or as percentage. In the latter case, 100% is the value provided by full-length wtNTAIL.
